# Lower Motoneuron Dysfunction Impacts Spontaneous Motor Recovery in Acute Cervical Spinal Cord Injury

**DOI:** 10.1089/neu.2022.0181

**Published:** 2023-04-28

**Authors:** Steffen Franz, Ute Eck, Christian Schuld, Laura Heutehaus, Marcel Wolf, Einar Wilder-Smith, Wilhelm Schulte-Mattler, Marc-André Weber, Rüdiger Rupp, Norbert Weidner

**Affiliations:** ^1^ Spinal Cord Injury Center, Heidelberg University Hospital, Heidelberg, Germany.; ^2^ Department of Neuroradiology, Heidelberg University Hospital, Heidelberg, Germany.; ^3^ Department of Neurology, Kantonsspital Lucerne, Lucerne, Switzerland.; ^4^ Department of Neurology, Inselspital Bern, University of Bern, Bern, Switzerland.; ^5^ Regensburg, Germany.; ^6^ Institute of Diagnostic and Interventional Radiology, Pediatric Radiology and Neuroradiology, Rostock University Medical Center, Rostock, Germany.

**Keywords:** axonal sprouting, clinical trials, denervation, nerve transfer surgery, neuroprostheses

## Abstract

Paresis after spinal cord injury (SCI) is caused by damage to upper and lower motoneurons (LMNs) and may differentially impact neurological recovery. This prospective monocentric longitudinal observational study investigated the extent and severity of LMN dysfunction and its impact on upper extremity motor recovery after acute cervical SCI. Pathological spontaneous activity at rest and/or increased discharge rates of motor unit action potentials recorded by needle electromyography (EMG) were taken as parameters for LMN dysfunction and its relation to the extent of myelopathy in the first available spine magnetic resonance imaging (MRI) was determined. Motor recovery was assessed by standardized neurological examination within the first four weeks (acute stage) and up to one year (chronic stage) after injury. Eighty-five muscles of 17 individuals with cervical SCI (neurological level of injury from C1 to C7) and a median age of 54 (28–59) years were examined. The results showed that muscles with signs of LMN dysfunction peaked at the lesion center (Χ^2^ [2, *n* = 85] = 6.6, *p* = 0.04) and that the severity of LMN dysfunction correlated with T2-weighted hyperintense MRI signal changes in routine spine MRI at the lesion site (Spearman ρ = 0.31, *p* = 0.01). Muscles exhibiting signs of LMN dysfunction, as indicated by pathological spontaneous activity at rest and/or increased discharge rates of motor unit action potentials, were associated with more severe paresis in both the acute and chronic stages after SCI (Spearman ρ acute = -0.22, *p* = 0.04 and chronic = -0.31, *p* = 0.004). Moreover, the severity of LMN dysfunction in the acute stage was also associated with a greater degree of paresis (Spearman ρ acute = -0.24, *p* = 0.03 and chronic = -0.35, *p* = 0.001). While both muscles with and without signs of LMN dysfunction were capable of regaining strength over time, those without LMN dysfunctions had a higher potential to reach full strength. Muscles with signs of LMN dysfunction in the acute stage displayed increased amplitudes of motor unit action potentials with chronic-stage needle EMG, indicating reinnervation through peripheral collateral sprouting as compensatory mechanism (Χ^2^ [1, *n* = 72] = 4.3, *p* = 0.04). Thus, LMN dysfunction represents a relevant factor contributing to motor impairment and recovery in acute cervical SCI. Defined recovery mechanisms (peripheral reinnervation) may at least partially underlie spontaneous recovery in respective muscles. Therefore, assessment of LMN dysfunction could help refine prediction of motor recovery after SCI.

## Introduction

Outcomes in cervical spinal cord injury (SCI) are heterogeneous.^1^ The degree of spontaneous recovery over time can be predicted by assessing initial completeness of sensorimotor dysfunction, albeit to a limited extent.^2–4^ With more comprehensive approaches relying on a variety of descriptive variables such as motor scores, sensory scores, age, neurological level of injury, a more precise outcome prediction beyond the America Spinal Injury Association impairment scale (AIS) can be achieved.^1,5,6^ These prediction rules, however, have not yet incorporated pathophysiological underpinnings. In this context, the relevance of lower motoneuron (LMN) dysfunction—widely known to occur after SCI—has yet to be determined.^7^

Paresis as a result of SCI is primarily caused by long descending pathway disruption such as the corticospinal tract, commonly referred to as the upper motoneuron. In particular, in cervical and lumbar SCI, both upper motoneuron *and* LMN damage or dysfunction contribute to paresis to a varying extent.^8^ Routine clinical assessment of voluntary motor strength, however, which is conducted according to the International Standards for Neurological Classification of Spinal Cord Injury (ISNCSCI),^9^ does not allow discrimination between upper and LMN damage, particularly early after injury. Without structurally intact LMNs at and below the level of injury, spared long-axon motor tracts will not be able to maintain a functioning neural pathway to exert voluntary motor control.^10,11^

Cross-sectional investigations have already reported signs of LMN dysfunction in human SCI at and remote from the injury site during chronic stages.^12–19^ None of these studies, however, focused on the spatial extent or impact of LMN dysfunction on motor recovery after acute cervical SCI. Only one study analyzed signs of LMN impairment in a subset of participants with acute SCI, applying nerve conduction studies of the median and ulnar nerves—an approach that does not investigate specific LMN pools representing defined segmental levels.^15^

To date, needle electromyography (nEMG) is considered the gold standard clinical routine assessment to detect signals of LMN dysfunction recorded from muscles that are innervated by corresponding LMN pools.^20^ This requires confounding factors, such as peripheral nerve injuries or myopathies, to be ruled out. In the acute stage, the parameters “pathological spontaneous activity at rest” (PSA) and increased discharge rates of “motor unit action potentials” (MUAPs) can be used to indicate dysfunction or loss of motor units/LMNs. In contrast, during the chronic stage, high amplitudes of MUAPs are indicative of subsequent compensatory reinnervation (peripheral collateral sprouting) and can be detected after LMN damage.^21–23^

Studies reporting multi-segmental innervation of muscles in cervical and lumbar regions have already discussed the clinical implications of LMN damage after localized injuries to the spinal cord or individual nerve roots.^24,25^ In addition, experiences from relevant studies regarding functional electrical stimulation (FES)-based neuroprostheses and nerve transfer surgeries have confirmed that LMN dysfunction is a relevant limiting factor.^14,26^

Hence, the mentioned aspects imply that LMN dysfunction may play a more important role in functional recovery after cervical SCI than previously assumed. It is therefore clinically relevant to investigate LMN dysfunction already at an acute stage after SCI and evaluate its impact on motor recovery over the course of time. The present observational study primarily aimed to address these questions. These findings will not only further the understanding of (motor) recovery processes during comprehensive SCI care but also help predict responsiveness to the innovative therapeutic strategies mentioned above, all of which rely on the integrity of LMNs.^14,26,27^

For these reasons, individuals with acute cervical SCI prospectively underwent standardized neurological assessments according to ISNCSCI, nerve conduction studies, and nEMG. In addition, nEMG findings related to LMN dysfunction were correlated retrospectively with myelopathy as depicted in early routine spine MRI. This approach aimed to evaluate early MRI as an additional clinically relevant readout for indications of structural damage and to further refine the estimation of the rostrocaudal extent and pattern of affected LMNs.

## Methods

### Study design and setting

**FIG. 1.**
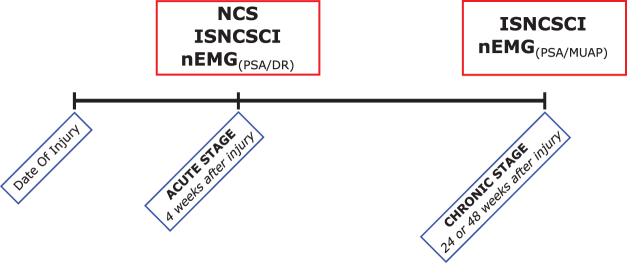
Study timeline. Red boxes denote medical diagnostics and the clinical assessment as performed according to the International Standards for Neurological Classification of SCI (ISNCSCI). Blue boxes denote the scheduled visits (acute stage = inpatient treatment; chronic stage = outpatient care). While for the needle electromyography (nEMG) at the acute stage, pathological spontaneous activity at rest (PSA) and increased discharge rates (DR) were assessed, the nEMG at the chronic stage aimed to analyze PSA and motor unit action potential (MUAP) amplitudes. Nerve conduction studies (NCS) were only performed at the acute stage. Color image is available online.

This prospective monocentric longitudinal, observational cohort study was approved by the ethics committee of Heidelberg University (S-516-2011) and registered in the German Clinical Trials Registry (Registry-no. DRKS00006258). Before enrollment, written informed consent was obtained from all study participants. Patients were recruited from 2012 to 2016. The time schedule for clinical examinations was derived from the European Multicenter Study about SCI (EMSCI).^28^ An illustration of the course of the study and related examinations is given in Figure 1.

### Participants

**FIG. 2.**
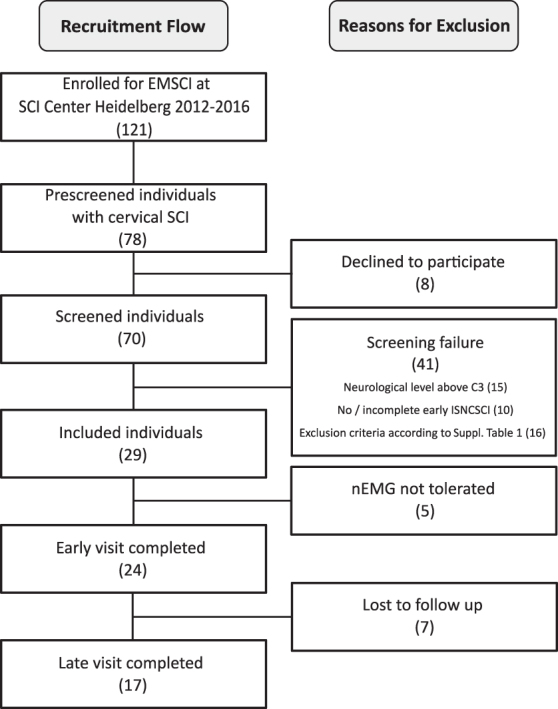
Recruitment flow chart. EMSCI, European Multicenter Study about Spinal Cord Injury; SCI, spinal cord injury; ISNCSCI, International Standards for Neurological Classification of Spinal Cord Injury; nEMG, needle electromyography.

Individuals with traumatic or ischemic SCI of any severity (AIS A–D) were identified by consecutive sampling and considered eligible primarily if they presented a neurological level of injury (NLI) between C3–C8. Because of a high number of screening failures (Fig. 2), patients presenting with a NLI above C3 were also considered for inclusion if they had stable spontaneous respiration. Sample size was thus based on the average number of admissions. The detailed inclusion and exclusion criteria included the EMSCI and other additional parameters, all of which are listed in Supplementary Table S1.^28^ Administered medication that could potentially interfere with motor recovery was recorded. Three individuals received baclofen, another three received opioids, and none received benzodiazepines.

### Standardized clinical assessment

Neurological condition was assessed by trained ISNCSCI assessors to ensure high quality standards.^29,30^ Participants were examined in accordance with the EMSCI time schedule at least once in the acute stage (16–40 days) *and* once in the chronic stage (150–186 or 300–546 days) after SCI (Fig. 1).^31^ Motor function of the upper extremity key muscles was assessed as muscle strength graded on a six-level scale.^32^ According to the ISNCSCI rules, the motor level follows the sensory level rostral to C5, which is the most rostral myotome represented by a key upper extremity muscle.

Findings at the motor level and one segment caudal to it are referred to as “at-level.” Segments rostral to the motor level are termed “above-level,” while those more than one segment caudal to the defined motor level are considered as “below-level.”

### Calculation of relative loss of motor function in the upper extremities

The relative loss of motor function in the upper extremities was based on the maximum achievable sum scores below the level of injury and the examined sum scores. It was determined according to the following formula:

$$\frac{\left[UEMSMAX\right]-\left[UEMSEXAM\right]}{\left[UEMSMAX\right]}x100=\left[LoMF\right]$$

UEMS_MAX stands for the total maximum below-(motor) level upper extremity sum motor score on both sides of the body, UEMS_EXAM for the actual examined below-(motor) level upper extremity sum motor score on both sides of the body at the acute stage, and LoMF for the relative loss of motor function.

### Assessment of LMN integrity

Needle EMG was chosen to detect signs of LMN dysfunction and performed in five defined key muscles of the dominant arm (C5 to T1) at three different examination sites by two board-certified neurologists.^33^ With one exception, only upper extremity key muscles according to ISNCSCI were chosen.^9^ Instead of the flexor digitorum profundus muscle, the abductor pollicis brevis muscle, representing the spinal segment C8, was investigated because of better accessibility for nEMG.^34^ The biceps brachii muscle (C5), the extensor carpi radialis longus muscle (C6), the triceps brachii muscle (C7), and the abductor digiti minimi muscle (T1) were additionally examined to provide a comprehensive mapping of the segmental innervation in the cervical spinal cord.

Care was taken to rule out other causes of nEMG abnormalities such as neuromuscular diseases, polyneuropathy, brachial plexus injury, and any other known lesion of the peripheral nervous system (Supplementary Table 1): (a) past medical history, (b) early MRI to determine apparent cervical nerve root compression (please see next section “MRI processing”), (c) motor nerve conduction studies of the ulnar nerve (EMSCI protocol). In case of pathological findings, sensory nerve conduction studies of the ulnar nerve were added to exclude a subclinical peripheral nerve lesion (Supplementary Table S2).

**Table 1.** Characteristics at the Acute and Chronic Stage According to the International Standards for Neurological Classification of Spinal Cord Injury

				Investigated side of the body					Contralateral side of the body				
ID	AIS	NLI^a^	Cause	Acute ML	Acute UEMS^b^	Chronic UEMS^b^	Acute MS^c^	Chronic MS^c^	Acute ML	Acute UEMS^b^	Chronic UEMS^b^	Acute MS^c^	Chronic MS^c^
01	A	C3	T	C3	8	12	2 (0-2)	1 (1-5)	C3	6	7	1 (0-1)	1 (0-2)
02	C	C4	T	C4	4	23	1 (0-1)	5 (4-5)	C4	6	21	1 (1-2)	4 (4-4)
03	D	C4	T	C6	14	20	2 (1-5)	4 (4-4)	C6	11	18	1 (0-5)	3 (3-4)
04	D	C2	T	C2	4	24	1 (1-1)	5 (5-5)	C4	13	23	3 (2-3)	5 (4-5)
05	D	C5	T	C7	17	23	4 (2-5)	5 (4-5)	C6	14	22	4 (1-4)	4 (4-5)
06	D	C4	I	C5	10	14	2 (0-4)	4 (0-5)	C6	10	14	2 (0-3)	4 (1-4)
07	C	C2	I	C2	10	23	2 (2-2)	5 (4-5)	C2	7	21	1 (1-2)	4 (4-4)
08	C	C1	T	C1	0	16	0 (0-0)	4 (3-4)	C1	0	14	0 (0-0)	4 (1-4)
09	C	C4	T	C4	3	19	0 (0-1)	4 (4-5)	C5	5	13	1 (0-1)	3 (1-4)
10	B	C4	T	C6	14	15	4 (1-4)	4 (1-5)	C5	8	12	1 (0-3)	3 (0-4)
11	D	C4	I	C6	15	18	4 (1-4)	4 (3-4)	C7	24	23	5 (5-5)	5 (4-5)
12	C	C3	T	C3	2	4	0 (0-1)	1 (1-1)	C4	3	2	1 (0-1)	0 (0-1)
13	D	C4	T	C4	6	11	1 (1-1)	2 (1-4)	C6	18	22	3 (3-4)	4 (4-5)
14	D	C4	T	C6	14	21	4 (0-5)	5 (4-5)	C8	21	23	5 (3-5)	5 (4-5)
15	B	C7	T	C7	18	21	4 (3-5)	5 (4-5)	C7	20	22	4 (4-5)	5 (4-5)
16	C	C4	T	C6	10	20	1 (0-4)	4 (4-4)	C5	7	21	1 (0-2)	4 (4-4)
17	D	C3	T	C3	22	24	5 (4-5)	5 (5-5)	C7	21	23	4 (4-5)	5 (4-5)

AIS, American Spinal Injury Association impairment scale; NLI, neurological level of injury; Cause, cause of injury; ML, motor level; UEMS, upper extremity motor score; MS, motor score; T, traumatic; I, ischemic.

^a^ Defined as the most caudal segment that still has normal function on both sides of the body for both sensory perception and motor function.

^b^ Calculated as sum of all scores for upper extremity MS from C5 to T1 on a given side of the body.

^c^ Presented as median of the key muscles C5 to T1 on a given side of the body. Interquartile ranges are given in parentheses.

**FIG. 3.**
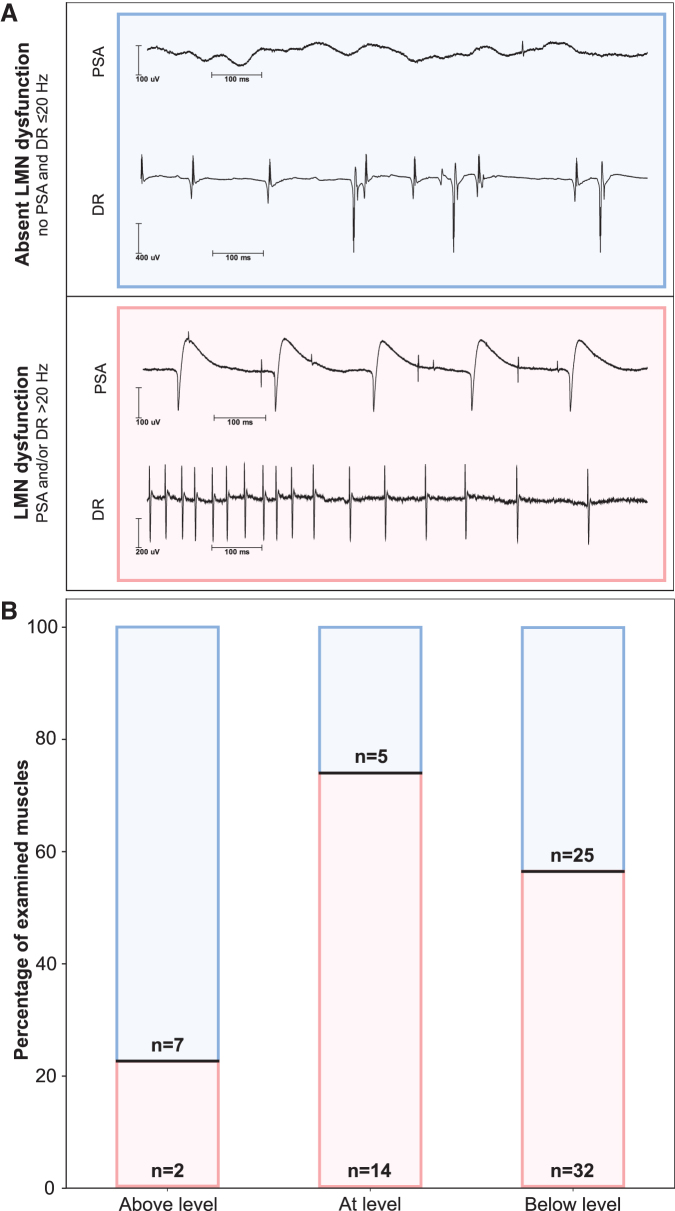
Rostrocaudal distribution of lower motoneuron (LMN) dysfunction. (**A**) Representative data acquired by needle electromyography (nEMG) comprising normal findings (blue box) and evidence of LMN dysfunction (red box). Evidence of LMN dysfunction is depicted by the detection of pathological spontaneous activity (PSA) or increased discharge rates (DR). (**B**) Needle-EMG findings of all examined muscles (*n* = 85) are illustrated in relation to the motor level (x-axis), which is defined as the most caudal myotome with at least antigravity strength and normal motor function rostral to it according to the International Standards for Neurological Classification of SCI. “Above” summarizes all segments at least one level rostral to the motor level. “At” stands for those segments that are located at or no more than one segment below the motor level, while “Below” is defined by all the segments that are more than one level below the motor level. The relative distribution of muscles with (red) and without (blue) LMN dysfunction as percentage of all examined muscles is shown in each of the three bars (y-axis). Absolute numbers (*n*) of muscles are given in respective sections of the bars. Color image is available online.

To assess dysfunction of LMNs, PSA and discharge rates of MUAPs were determined and evaluated as described previously (Fig. 3A).^21–23^ To eliminate false positives, amplitudes of action potentials below 20 μV were excluded to minimize the risk of mistaking artifacts or other volume-conducted activity for PSA. Analysis and interpretation of both PSA and MUAP were based on a decomposed train of five matching potentials in accordance with published standards.^22,35–37^

The PSA represent discharges of denervated or damaged (e.g., myopathy) single muscle fibers.^21,23^ Increased discharge rates are caused by a loss of motor units.^22^ Both PSA and increased discharge rates indicate LMN dysfunction but may occur independently. Therefore, LMN dysfunction was assumed when PSA and/or discharge rates of MUAPs >20 Hz were detectable. Discharge rates of MUAPs were handled as a binary classifier (≤20 Hz/>20 Hz). The severity of PSA was graded semi-quantitatively on a four-level ordinal scale from 0 to 3 as described previously.^38^ The PSA was correlated with the six-level ordinal-scaled motor function of the five key muscles (C5 to T1) defined by ISNCSCI.^9^

Based on relevant literature and broadly accepted routine practice, muscles displaying MUAP amplitudes ≥2 mV (observed in three different MUAPS per muscle; rise time >0.1 msec) were considered to have undergone reinnervation by collateral sprouting as a compensatory mechanism at the chronic stage nEMG.^39,40^ Needle EMGs were sampled with 10 kHz and decompositioned in a nEMG recording device (Schwarzer Topas, Natus, Munich, Germany). Recordings were done under acoustic control with the following settings: skin temperature >30°C, (bandpass) filter 5 Hz to 5 kHz, amplification 50 μV/Div (PSA)/0.1–5.0 mV/Div (MUAP), and sweep speed to 10 msec/Div.

### MRI processing

The anatomical T2-weighted MRIs were acquired on 1.5T and 3T scanners (Siemens: 1xAera, 2xAvanto, 2xEspree, 8xSymphony, 2xTrio, 2xVerio) immediately after admission to the primary care hospital and—if applicable—before spine surgery. The MRI parameters were as follows (see also Supplementary Table S3): slice thickness for the sagittal plane 17x 3.0 mm (turbo spin echo sequence), and for the axial plane 1x 2.0 mm (gradient echo sequence), 11x 3.0 mm (10x turbo spin echo and 1x gradient echo sequence), and 5x 4.0 mm (turbo spin echo sequence).

**FIG. 4.**
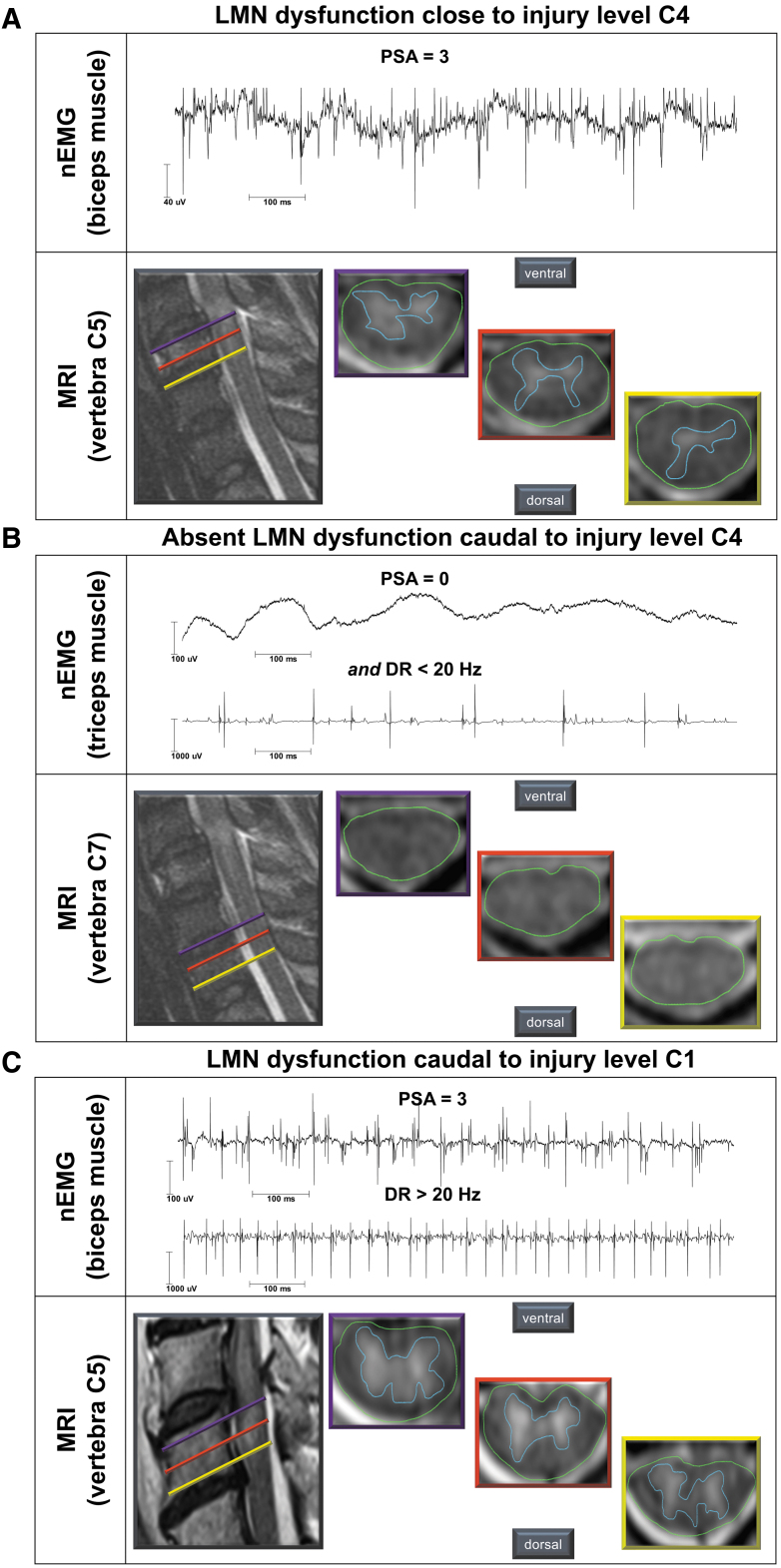
Typical findings of needle electromyography (nEMG) and routine magnetic resonance imaging (MRI). (**A**) Needle-EMG shows severe (grade 3) pathological spontaneous activity (PSA) in the biceps muscle—the key muscle for segment C5 according to the International Standards for Neurological Classification of SCI in an individual with a C4 lesion level. The PSA was characterized by a full interference pattern of abundant PSA in all examined areas. The MRI shows marked myelopathy at the corresponding segmental level of vertebral body C5. (**B**) At C7 level of the same individual—three segments caudal to the lesion level (C4)—nEMG and MRI are normal. (**C**) The lower motoneuron (LMN) dysfunction (severe PSA and increased discharge rates [DR] of motor unit action potentials) identified four segments caudal (biceps muscle) to the lesion level C1 in an individual with longitudinally extensive myelopathy at the corresponding level of vertebra C5. Extensive myelopathy was likely caused by an intraspinal hematoma, which compressed the spinal cord beyond the lesion level. Color image is available online.

The T2-weighted hyperintense MRI signal changes at the spinal cord lesion site were considered indicative of myelopathy and associated structural damage. Subsequently, the extent of myelopathy was compared with the nEMG findings (Fig. 4) and evaluated for its value in estimating motor recovery. The extent of myelopathy detected by MRI was expressed as percentage of the cross-sectional area of the spinal cord in each segment.

The MRI analysis was performed by a board-certified neuroradiologist and a board-certified neurologist using conventional open-source processing software (DICOM viewer OsiriX Lite, Pixmeo SARL, Bernex, Switzerland). Cervical neuroforamina were analyzed to exclude compression of nerve roots. The spinal cord segmental level (C1-T1) was assigned as a reference to the respective vertebral body^25,41^: three T2-weighted MRI axial scans per cervical segment were analyzed as a percentage of the cross-sectional area of the spinal cord. For this, the relevant sections of each cervical segment were derived from the scan closest to the cover plate, the mid-level, and the base plate of the respective vertebral body.

The area showing hyperintense signal changes representing myelopathy of the spinal cord was marked and normalized by the total cross-sectional area of the spinal cord (Fig. 4). Subsequently, a mean value was calculated from the three obtained percentages of myelopathy for each segment.

Finally, the extent of myelopathy for each segment was averaged over both examiners. The following formula summarizes the workflow:

$$\begin{array}{c} myelopathy\left(segment\right)=\frac{1}{2}\sum_{examiner}^{SF,MW}\\ \left(\frac{1}{3}\sum_{location}^{cover,middle,base}\frac{myelopathy\left(examiner,location,segment\right)}{crosssection\left(examiner,location,segment\right)}\right). \end{array}$$

### Quantitative variables and grouping

The presence of LMN dysfunction was classified with a binary variable as either *negative* or *positive*. It was considered *positive* in case of PSA >0 and/or discharge rates of single MUAP >20 Hz were detected. For semiquantitative evaluation of PSA, a grading of 2 and 3 reflected moderate to severe LMN dysfunction, whereas a grade of 1 was considered mild LMN dysfunction. Needle EMG findings were grouped by the segmental distance from the ISNCSCI motor level. Negative numbers represent segments rostral to the lesion, whereas positive numbers stand for segments caudal to the lesion.

### Statistical analyses

Data were processed, analyzed, and visualized using the Python Data Science Stack, i.e., pandas (data processing),^42^ matplotlib (visualization),^43^ and scipy (statistics).^44^

The Χ^2^-test was used to test the distribution of LMN dysfunction in the above-level, at-level, and below-level grouping, the association of LMN dysfunction with high amplitudes of MUAPS and motor recovery, as well as subgroup analysis (AIS A/B versus AIS C/D).

Spearman's ρ rank correlation coefficient was used to test the association between ordinally distributed variables such as the ISNCSCI motor score or the PSA. Association was interpreted as slight (r ≥ 0.1), moderate (r ≥ 0.3), or strong (r ≥ 0.5).^45^ Categorical data are presented as median and related interquartile range (IQR) or both 25^th^ and 75^th^ percentiles. Exact *p* values are reported with α <0.05 as threshold for significance.

## Results

Within 51 months, 121 individuals met the EMSCI inclusion criteria. Of these, 78 presented with cervical SCI and were asked to participate in a pre-screening to check for eligibility. Eight individuals declined, and the remaining 70 were screened. Twenty-nine individuals were then included in the study, of whom five did not tolerate nEMG. Of the remaining 24 participants, 17 (15 males, 2 females) with a median age of 54 (28–59) years completed the whole study protocol including an assessment in both the acute and the chronic stage of SCI (Fig. 2). Fourteen had traumatic and three had ischemic SCI. One individual presented a complete injury (AIS A) and two others a sensory incomplete lesion (AIS B). Fourteen study participants had a motor incomplete injury (6x AIS C and 8x AIS D).

Participants were characterized by a relative loss of motor function in the upper extremities as follows: entire cohort (AIS A to AIS D) = 71.2%; motor complete individuals (AIS A/B) = 71.4%; motor incomplete individuals (AIS C/D) = 67.4% (Supplementary Table S4).

Needle EMG was performed in the acute stage 54 (42–57) days and in the chronic stage 361 (301–375) days post-injury. While all 17 participants agreed to each key muscle examination C5–T1 (*n* = 85) during the acute stage, two of the 17 participants declined nEMG in the chronic stage. In addition, one subject refused nEMG in three key muscles (C7, C8, and T1). Therefore, 72 muscles were assessed in the chronic stage.

Myelopathy was evaluated retrospectively based on routine spine MRI conducted no later than 16 days after injury (1.6 ± 4.0 days). Analysis of neuroforamina revealed no (apparently persistent) compression of nerve roots in any of the participants. In 10 participants, the axial scans of the MRI did not cover all relevant segments leading to missing MRI data (*n* = 69) for 16 segments, with one segment missing in six individuals, two segments missing in two, and three segments missing in two additional participants. Clinical examinations (ISNCSCI) were performed at 28 (23–33) days after injury in the acute stage and 361 (301–375) days after injury in the chronic stage (Table 1).

### Distribution and severity of LMN dysfunction

In the acute stage after SCI, signs of LMN dysfunction were found in 56.5% of all examined muscles, with the highest ratio in muscles at-level (73.7%), followed by 56.1% of the examined muscles below-level and 22.2% of the muscles above-level (Χ^2^ [2, *n* = 85] = 6.6, *p* = 0.04; Fig. 3B).

**FIG. 5.**
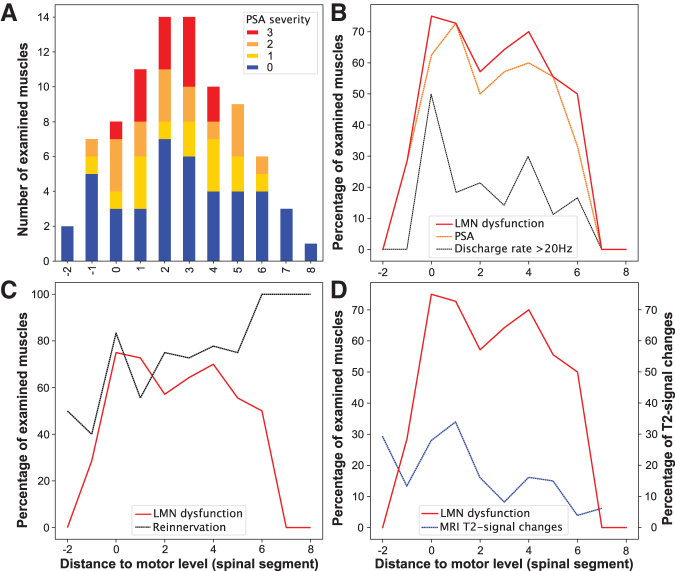
Segmental distribution of lower motoneuron (LMN) dysfunction. (**A-D**) x-axis: number of spinal segments rostral (minus) and caudal (plus) referenced to the motor level (0). (**A**) Examined muscles (absolute numbers; y-axis) showing increasing severity of pathological spontaneous activity (PSA) from 0–3 as an indicator of the severity of LMN dysfunction. Severity of PSA is represented in respective sections of the bars. (**B**) Ratio of muscles with LMN dysfunction of all examined muscles (percent; y-axis) for each segment illustrating the parameters PSA (orange dashed line), discharge rate >20 Hz (gray dotted line), and the resulting determination of LMN dysfunction (red solid line). (**C**) Segmental ratio (percent; y-axis) of muscles displaying LMN dysfunction (red solid line) and signs of reinnervation (motor unit action potentials >2 mV) after one year (gray dashed line). (**D**) Relative covering (percent, left y-axis) of hyperintense magnetic resonance imaging (MRI) T2-signal changes over the cross-sectional area of the spinal cord (blue dashed line) compared with the percentage (right y-axis) of LMN dysfunction (red solid line). Color image is available online.

Overall, severe LMN dysfunction, as determined by a PSA grade of 2 or 3, was found in 29 of all 85 (34%) muscles tested. Of all muscles at-level, 47% (*n* = 9/19) showed signs of severe PSA compared with 33% (*n* = 19/57) of all muscles more than one level below the motor level (Fig. 5A). Sixteen percent (*n* = 9/57) of muscles below-level displayed mild PSA. No acute signs of LMN dysfunction were detected in muscles more than six levels caudal to the motor level (*n* = 4). Only two of the nine muscles examined more than one level rostral to the motor level showed signs of LMN dysfunction. Accordingly, these findings could be confirmed by a significant negative association between PSA severity and increasing distance from the lesion site (Spearman ρ = -0.24, *p* = 0.04, *n* = 76).

A subgroup analysis revealed an association between LMN dysfunction and motor completeness of SCI (AIS A/B; *n* = 3),^46^ with a higher prevalence of muscles with signs of LMN dysfunction in participants with motor complete lesions (complete : incomplete = 80% : 51%, Χ^2^ [1, *n* = 85] = 4.1, *p* = 0.04). Segment-by-segment analysis revealed that the percentage of muscles with abnormal nEMG findings peaked around the lesion level and decreased over five segments caudal to the motor level (Fig. 5B).

As expected, overall PSA severity attenuated from a median of 1 to 0 over the first year after injury (*p* = 0.02). Of the 72 muscles in which nEMG was performed at both acute and chronic stages, 41 showed LMN dysfunction at the acute stage, 34 (83%) of which transitioned to display amplitudes of MUAPs ≥2 mV indicating collateral sprouting in the chronic stage (Χ^2^ [1, *n* = 72] = 4.3, *p* = 0.04). The segmental distribution of high MUAP amplitudes was relatively balanced at-level and up to six segments below (Fig. 5C). The high proportion of muscles exhibiting high amplitudes of MUAPs in the margin areas may largely be explained by a selection bias because of low sample sizes: only two muscles two segments above the motor level, one muscle eight segments and another three muscles seven segments below the motor level.

### LMN dysfunction and extent of myelopathy

An association of LMN dysfunction with the extent of myelopathy—i.e., MRI T2-signal changes (Spearman ρ = 0.26, *p* = 0.03, *n* = 69)—was found. Likewise, higher grades of PSA correlated moderately with the extent of myelopathy (Spearman ρ = 0.31, *p* = 0.01, *n* = 69; Fig. 4). Both the proportion of affected LMNs and myelopathy displayed a comparable segmental distribution pattern, with a peak around the injury site and a decrease in consecutive caudal segments (Fig. 5D).

### LMN dysfunction and recovery of motor function

**FIG. 6.**
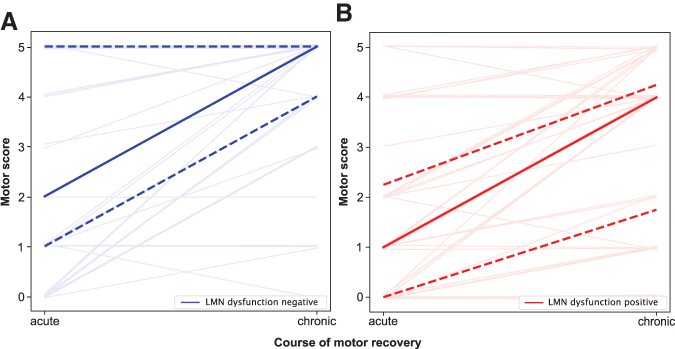
Lower motoneuron (LMN) dysfunction and motor recovery within the first year after spinal cord injury (SCI). (**A,B**) Y-axis: motor score from 0 to 5. X-axis: stage after SCI of the assessments (acute and chronic), median (bold line), 25^th^ and 75^th^ percentile (dashed lines), detail drawing of all examined muscles (faint lines). (**A**) Changes of muscle strength from acute to chronic stage (x-axis) within the first year after SCI in the investigated muscles without acute LMN dysfunction. (**B**) Changes of muscle strength from acute to chronic stage within the first year after SCI in the investigated muscles displaying acute LMN dysfunction. Color image is available online.

Motor scores of muscles with and without signs of dysfunctional LMNs both gained three motor-score-points (median) each between the acute and chronic stages. Muscles with signs of LMN dysfunction, however, maintained lower strength at both stages (median 1 motor-score-point) compared with muscles without signs of LMN dysfunction (Spearman ρ acute = -0.22, *p* = 0.04 and chronic = -0.31, *p* = 0.004; Fig. 6A,B; Table 2). The severity of LMN dysfunction, as determined by PSA grading, was also associated with lower motor scores at both the acute and the chronic stages (Spearman ρ acute = -0.24, *p* = 0.03 and chronic = -0.35, *p* = 0.001). Muscles referring to at-level myotomes displayed a slightly inferior gain in strength over time (1 motor-score-point) in case of LMN dysfunction (Table 2).

**Table 2.** Muscle Strength in Relation to Needle-Electromyography Findings with Reference to the Motor Level

		MS _acute_		MS _chronic_		
nEMG	Reference to ML	median	IQR	median	IQR	*n*
LMN dysfunction neg.	above-level	5.00	5.00-5.00	5.00	5.00-5.00	7
	at-level	4.00	4.00-5.00	5.00	5.00-5.00	5
	below-level	1.00	0.00-2.00	4.00	3.00-5.00	25
	at- and below-level	1.00	0.25-3.75	4.50	3.25-5.00	30
	total	2.00	1.00-5.00	5.00	4.00-5.00	37
						
LMN dysfunction pos.	above-level	5.00	5.00-5.00	4.50	4.25-4.75	2
	at-level	4.00	2.00-4.00	4.00	4.00-5.00	14
	below-level	1.00	0.00-1.00	4.00	1.00-4.00	32
	at- and below-level	1.00	0.00-2.00	4.00	1.25-4.00	46
	total	1.00	0.00-2.25	4.00	1.75-4.25	48

nEMG, needle electromyography; ML, motor level; MS, motor score; IQR, interquartile range; n, number of tested muscles; LMN, lower motoneuron.

## Discussion

In the present study, LMN dysfunction was associated with more severe paresis early on and predisposed to inferior motor recovery compared with muscles without signs of LMN dysfunction over a period of up to one year after injury. Chronic stage nEMG findings together with spine MRI findings suggest that the dysfunction is most likely caused by structural damage causing cell death/degeneration of LMNs.

The overall pattern of a relatively high proportion of muscles with signs of LMN dysfunction including a wide rostrocaudal spread has been described in previous studies, which are not directly comparable because of different methods (analysis of post-mortem human spinal cord) and different parameters chosen for quantification of EMG results.^12,18^ The previous EMG-based study described a discontinuous focus of LMN dysfunction several segments caudal to the injury site, whereas in the present study LMN dysfunction was pronounced at and around the injury site, with a more continuous spread over up to six segments caudally. Whether this discrepancy was solely related to the different methodological approaches cannot be conclusively determined.

Surprisingly, a high prevalence of muscles with signs of LMN dysfunction (80%) was not only found in individuals with motor complete lesions (AIS A and B), where a more widespread damage because of the presumed extensive structural damage was to be expected. Even in individuals with motor incomplete SCI (AIS C/D), 51% of investigated muscles displayed LMN dysfunction. Previous studies did not provide comparable data in this respect.^12,18^

A further interesting finding was the detection of LMN dysfunction in a small number of muscles (*n* = 7, 8.2%) representing clinically intact spinal cord segments at or above the motor level. Previously reported multi-segmental innervation of muscles likely explains this phenomenon, where innervation from a structurally affected segment targets a “key” muscle formally assigned to an above-level segment.^24,25,47^

### Which exact mechanisms are likely to have caused the observed widespread LMN dysfunction?

Permanent LMN dysfunction (at the acute and chronic stage) associated with the presence of structural damage detected by spine MRI suggest primary (impact of the trauma, ischemia)^18^ and/or secondary (inflammatory response)^48,49^ underlying causes in the majority of related muscles investigated, not only close to but also remote from the lesion site. In a subset of muscles investigated (17%), only transient LMN dysfunction (LMN dysfunction in the acute stage, no changes in the chronic stage) was detected, which may have been caused by transient changes such as edema.^50^

Mild LMN dysfunction (grade of PSA = 1) found in 16% of muscles related to spinal segments distant from the lesion center (below-level) was associated with rather slight impairment of voluntary motor function both in the acute and chronic stages of SCI. This may have been caused by transsynaptic degeneration.^51^ Whether such a mechanism really exists and how it exactly impacts clinical outcomes is still a matter of debate.^16,52–54^

### Why was LMN dysfunction associated with more severe paresis and potentially a lower likelihood of spontaneous motor recovery?

**FIG. 7.**
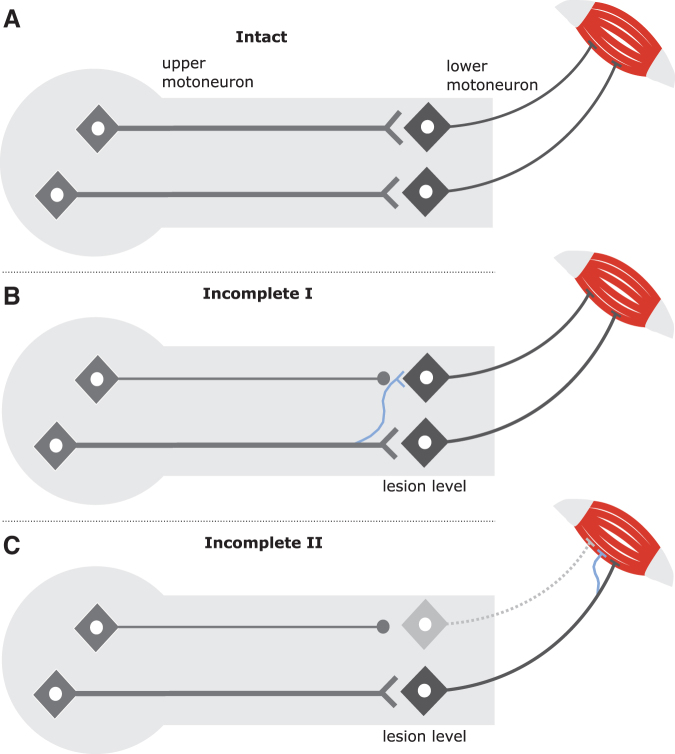
Mechanisms of structural rearrangements after spinal cord injury at the lesion level and close to corresponding muscles. (**A**) In the uninjured condition, long descending motor pathways—e.g. the corticospinal tract (upper motoneuron, UMN)—connect to lower motoneurons (LMNs) at the spinal segmental level, which in turn reach target muscles as a prerequisite for voluntary motor function. (**B**) After cervical spinal cord injury, one conceivable scenario is that only the axon derived from the UMN may be disrupted, while the LMN that originally served as the target structure is still intact. Consequently, spared long-axons of related motor tracts may form collateral sprouts (thin blue line) to reconnect with the LMN that was just deprived of the UMN. (**C**) The second scenario involves both a disrupted UMN and LMN. In such a case, potential regenerative processes could occur in the peripheral nervous system close to the myoneural junction, with spared LMNs forming collateral sprouts to reinnervate the muscle recently denervated because of the LMN damage that occurred. Color image is available online.

After cervical SCI, two different scenarios of neural damage can, in principle, lead to paretic muscles. In the first scenario, only the long-axon motor tracts (upper motoneuron/corticospinal tract) are disrupted. In the second scenario, both the long-axon motor tracts and the corresponding LMNs are damaged (Fig. 7). Isolated, direct LMN damage without disruption to the corresponding long-axon motor tracts is highly unlikely after SCI. In case of an isolated long-axon motor tract damage, the redundancy of innervation to maintain/recover motor control appears higher if only one system is disrupted.^55^

Accordingly, it seems plausible that a combined injury of upper motoneurons and LMNs leads to more pronounced paresis in muscles with signs of LMN dysfunction. Of course, any comparison of recovery rates in muscles with and without LMN dysfunction should be treated with caution because of the ordinal nature of motor scores and potential ceiling effects.

### Which mechanisms have led to recovery of motor control?

With respect to long-axon motor tract damage (CNS environment), several mechanisms have been discussed and identified to contribute to spontaneous motor recovery after SCI, the most prominent being collateral sprouting of uninjured axons and/or synaptic rearrangement at supraspinal and spinal levels (Fig, 7B).^55^ In case of combined long-axon motor tract disruption and LMN dysfunction, as occurs in cervical SCI, collateral sprouting of motor nerves in the peripheral nervous system—identified by high amplitudes of MUAPs indicative of reinnervation—may represent an additional compensatory mechanism for spontaneous recovery of motor function (Fig. 7C).^39^

The extent of spinal cord damage depicted by T2-weighted hyperintense MRI signal changes in cord parenchyma correlated with LMN dysfunction in respective myotomes. A more refined assessment of the ventral horn of the spinal cord gray matter, which contains the LMNs, was not feasible with reasonable confidence because of the variability of routine spine MRIs with respect to timing and imaging parameters. Moreover, the assessed MRIs were performed early after injury, where hyperintense signal changes in T2-weighted images are still vaguely defined^50^ and the affected spinal cord may have been compressed and distorted by dislocated spine fragments, further limiting reliable identification of the region of interest.

## Conclusions

The present study indicates that after a cervical SCI, LMN dysfunction is extensive and muscles with signs of LMN dysfunction lag behind muscles without such signs with respect to the maximum degree of motor control up to one year after injury. These findings may help to better predict motor recovery beyond routine comprehensive SCI care. The LMN dysfunction also challenges FES-based neuroprostheses and nerve transfer surgeries.^14,26^ Expanding on the findings of the present study, further important insights regarding force generation and fine motor skill performance in muscles with LMN dysfunction will be gained by introducing readouts from non-invasive electrical stimulation.^56^

The degree of LMN dysfunction as demonstrated in this study may represent a relevant confounding factor in clinical trials aiming for axon regeneration across the injury site.^57^ Even if successful axonal regrowth is achieved, the extent of motor recovery may be affected by dysfunctional LMNs.

## Supplementary Material

Supplemental data

Supplemental data

Supplemental data

Supplemental data
