# Supplementary material for: Lower Motoneuron Dysfunction Impacts Spontaneous Motor Recovery in Acute Cervical Spinal Cord Injury
Source: J Neurotrauma. 2023 Apr 28;40(9-10):862–75. doi: 10.1089/neu.2022.0181 (PMC10162119; doi:10.1089/neu.2022.0181)
Supplement: Supplemental data [file Supp_TableS1.docx]

**Supplementary Table 1. Inclusion and exclusion criteria.**

| Inclusion criteria |
| --- |
| - First clinical assessment possible within the first 8 weeks after incidence |
| - Full legal age |
| - Patient capable and willing to give written informed consent |
| - Single event traumatic or ischemic cervical spinal cord injury (motor level C3-C8) |
| Clinical conditions that led to an exclusion from the study |
| - Acute microbiological infection |
| - Cardio-vascular, pulmonary or metabolic diseases incl. diabetes mellitus |
| - Colonization with multi-resistant germs |
| - Dementia or other reasons that could compromise cooperation and or consent |
| - History of brachial plexus injury |
| - Neurologic diseases other than spinal cord injury (e.g. epilepsy, neuromuscular disease) |
| - Non-traumatic spinal cord injury, except for single ischemic event |
| - Polyneuropathy or other lesions of the peripheral nervous system |
| - Pregnancy |
| - Psychiatric disorders |
| - Severe traumatic brain injury |
| - Soft tissue disruption or open wounds |
| - Underlying oncologic disease |
| - Ventilator dependence |
