# Supplementary material for: Lower Motoneuron Dysfunction Impacts Spontaneous Motor Recovery in Acute Cervical Spinal Cord Injury
Source: J Neurotrauma. 2023 Apr 28;40(9-10):862–75. doi: 10.1089/neu.2022.0181 (PMC10162119; doi:10.1089/neu.2022.0181)
Supplement: Supplemental data [file Supp_TableS2.docx]

**Supplementary Table 2** Nerve conduction studies of the ulnar nerve on the arm being examined in each participant

|  |  | **Motor Neurography** | | **Sensory Neurography** | |
| --- | --- | --- | --- | --- | --- |
| **ID** | SOB | NCV (m/s) | AMP (mV) | NCV (m/s) | AMP (µV) |
| **01** | r | 50.0 | **4.1** | 55.6 | 12.8 |
| **02** | r | 60.5 | 10.9 |  |  |
| **03** | l | 53.3 | 6.5 |  |  |
| **04** | l | 60.8 | 13.5 |  |  |
| **05** | r | 54.8 | 10.3 |  |  |
| **06** | r | 50.0 | **1.0** | 59.2 | 27.5 |
| **07** | r | 59.0 | 6.0 |  |  |
| **08** | r | 57.9 | 6.9 |  |  |
| **09** | r | 51.0 | 6.6 |  |  |
| **10** | r | 48.0 | **0.2** | 57.2 | 32.9 |
| **11** | r | 57.3 | 10.2 |  |  |
| **12** | r | 54.1 | 6.8 |  |  |
| **13** | r | 57.1 | 7.7 |  |  |
| **14** | r | 51.0 | **3.7** | 59.0 | 40.2 |
| **15** | l | 53.8 | **0.3** | 53.6 | 19.4 |
| **16** | r | 54.8 | 14.0 |  |  |
| **17** | r | 54.5 | 7.6 |  |  |
| *Abbreviations:* amplitude (AMP); nerve conduction velocity (NCV); side of body (SOB); | | | | | |
