# Supplementary material for: Lower Motoneuron Dysfunction Impacts Spontaneous Motor Recovery in Acute Cervical Spinal Cord Injury
Source: J Neurotrauma. 2023 Apr 28;40(9-10):862–75. doi: 10.1089/neu.2022.0181 (PMC10162119; doi:10.1089/neu.2022.0181)
Supplement: Supplemental data [file Supp_TableS3.docx]

**Supplementary Table 3** Technical specifications of MRI studies sorted by date, study participants and locations.

|  |  |  |  |  | **T2 sagittal** | |  | **T2 axial** |  |  |
| --- | --- | --- | --- | --- | --- | --- | --- | --- | --- | --- |
| **ID** | **Date of MRI** | **Hospital** | **Scanner** | **Field strength**  **in T** | **Sequence type** | **Slice thickness**  **in mm** | **TR/TE**  **in ms** | **Sequence type** | **Slice thickness**  **in mm** | **TR/TE**  **in ms** |
| **1** | 23.02.2012 | University Hospital Frankfurt | Siemens Espree | 1.5 T | TSE | 3.0 | 4000/85 | TSE | 4.0 | 5140/130 |
| **2** | 07.06. 2012 | University Hospital Heidelberg | Siemens Verio | 3.0 T | TSE | 3.0 | 3964/108 | TSE | 3.0 | 5010/114 |
| **3** | 22.08. 2012 | University Hospital Mainz | Siemens Espree | 1.5 T | TSE | 3.0 | 3810/92 | TSE | 3.0 | 4800/88 |
| **4** | 21.10. 2012 | University Hospital Frankfurt | Siemens Avanto | 1.5 T | TSE | 3.0 | 4292/87 | TSE | 3.0 | 5771/96 |
| **5** | 03.05. 2013 | University Hospital Heidelberg | Siemens Verio | 3.0 T | TSE | 3.0 | 3500/108 | GRE | 2.0 | 882/22 |
| **6** | 30.04. 2013 | University Hospital Heidelberg | Siemens Trio | 3.0 T | TSE | 3.0 | 3420/115 | TSE | 3.0 | 6930/108 |
| **7** | 14.07. 2013 | University Hospital Mainz | Siemens Avanto | 1.5 T | TSE | 3.0 | 3500/84 | GRE | 3.0 | 618/17 |
| **8** | 24.07. 2013 | University Hospital Erlangen | Siemens Aera | 1.5 T | TSE | 3.0 | 3090/81 | TSE | 3.0 | 5050/84 |
| **9** | 01.11. 2013 | University Hospital Würzburg | Siemens Symphony | 1.5 T | TSE | 3.0 | 3740/124 | TSE | 3.0 | 3650/97 |
| **10** | 04.11. 2013 | University Hospital Mannheim | Siemens Trio | 3.0 T | TSE | 3.0 | 4340/103 | TSE | 3.0 | 9640/94 |
| **11** | 11.05. 2014 | University Hospital Saarland | Siemens Symphony | 1.5 T | TSE | 3.0 | 3660/97 | TSE | 3.0 | 6852/112 |
| **12** | 11.12. 2014 | University Hospital Würzburg | Siemens Symphony | 1.5 T | TSE | 3.0 | 5200/115 | TSE | 4.0 | 6390/177 |
| **13** | 13.05. 2015 | University Hospital Würzburg | Siemens Symphony | 1.5 T | TSE | 3.0 | 3740/124 | TSE | 4.0 | 3500/95 |
| **14** | 19.06. 2015 | University Hospital Würzburg | Siemens Symphony | 1.5 T | TSE | 3.0 | 4400/115 | TSE | 3.0 | 3500/95 |
| **15** | 01.09. 2015 | Hospital Darmstadt | Siemens Symphony | 1.5 T | TSE | 3.0 | 4750/102 | TSE | 3.0 | 5930/109 |
| **16** | 18.09. 2015 | University Hospital Würzburg | Siemens Symphony | 1.5 T | TSE | 3.0 | 4390/124 | TSE | 4.0 | 3500/95 |
| **17** | 02.10. 2015 | Hospital Darmstadt | Siemens Symphony | 1.5 T | TSE | 3.0 | 5870/102 | TSE | 4.0 | 8470/109 |
| *Abbreviations:* gradient echo sequence (GRE); magnetic resonance imaging (MRI); Tesla (T); echo time (TE); repetition time (TR); turbo spin echo sequence (TSE) | | | | | | | | | | |
|  |  |  |  |  |  |  |  |  |  |  |
