# Supplementary material for: Lower Motoneuron Dysfunction Impacts Spontaneous Motor Recovery in Acute Cervical Spinal Cord Injury
Source: J Neurotrauma. 2023 Apr 28;40(9-10):862–75. doi: 10.1089/neu.2022.0181 (PMC10162119; doi:10.1089/neu.2022.0181)
Supplement: Supplemental data [file Supp_TableS4.docx]

**Supplementary Table 4** Loss of motor function in the acute stage of SCI, presented by comparison of the theoretical maximum and effectively examined motor sum scores below the motor level on a given side

|  |  |  |  | **Investigated side of the Body** | | | **Contralateral side of the body** | | |
| --- | --- | --- | --- | --- | --- | --- | --- | --- | --- |
| **ID** | **AIS** | **NLI**^a^ | **Cause** | **Acute stage** | **Maximum below ML** | **Acute stage**  **below ML** | **Acute stage** | **Maximum below ML** | **Acute stage**  **below ML** |
|  |  |  |  | **ML** | **UEMS_MAX**^b^ | **UEMS_EXAM^c^** | **ML** | **UEMS_MAX**^b^ | **UEMS_EXAM**^c^ |
| **1** | A | C3 | T | C3 | 25 | 8 | C3 | 25 | 7 |
| **2** | C | C4 | T | C4 | 25 | 4 | C4 | 25 | 21 |
| **3** | D | C4 | T | C6 | 15 | 4 | C6 | 15 | 6 |
| **4** | D | C2 | T | C2 | 25 | 4 | C4 | 25 | 13 |
| **5** | D | C5 | T | C7 | 10 | 2 | C6 | 15 | 4 |
| **6** | D | C4 | I | C5 | 20 | 10 | C6 | 15 | 0 |
| **7** | C | C2 | I | C2 | 25 | 10 | C2 | 25 | 7 |
| **8** | C | C1 | T | C1 | 25 | 0 | C1 | 25 | 0 |
| **9** | C | C4 | T | C4 | 25 | 3 | C5 | 20 | 0 |
| **10** | B | C4 | T | C6 | 15 | 4 | C5 | 20 | 3 |
| **11** | D | C4 | I | C6 | 15 | 5 | C7 | 10 | 9 |
| **12** | C | C3 | T | C3 | 25 | 2 | C4 | 25 | 3 |
| **13** | D | C4 | T | C4 | 25 | 6 | C6 | 15 | 8 |
| **14** | D | C4 | T | C6 | 15 | 4 | C8 | 5 | 1 |
| **15** | B | C7 | T | C7 | 10 | 3 | C7 | 10 | 5 |
| **16** | C | C4 | T | C6 | 15 | 0 | C5 | 20 | 2 |
| **17** | D | C3 | T | C3 | 25 | 22 | C7 | 10 | 6 |
| ^a^Defined as the most caudal segment that still has normal function on both sides of the body for both sensory perception and motor function.  ^b^Calculated as maximum achievable score for upper extremity MS in testable myotomes (C5-T1) from one segment below the ML on the given side of the body.  ^c^Calculated as sum of all examined MS in each testable myotome (C5-T1) from one segment below the ML on the given side of the body.  *Abbreviations:* ASIA impairment scale (AIS); cause of injury (Cause); examined value (EXAM); ischemic (I); maximum value (MAX); motor level (ML); motor score (MS); neurological level of injury (NLI); traumatic (T); upper extremity motor score (UEMS). | | | | | | | | | |
